#  Marital Satisfaction in Refereed Women to Gorgan Health ‎Centers 

**Published:** 2016-07

**Authors:** Hashem Heshmati, Nasser Behnampour, Samira Arabameri, Samane Khajavi, Nooshin Kohan

**Affiliations:** 1 Department of Public Health, Faculty of Health, Torbat Heydariyeh University of Medical Sciences, Torbat ‎Heydariyeh, Iran.‎; 2 Department of Public Health, Faculty of Health, Golestan University of Medical Sciences, Gorgan, Iran.‎; 3 Department of Management, Golestan Payam-e-Noor University, Gorgan, Iran.; 4 Department of Medical Education, School of Medicine, Tehran University of Medical Sciences, Tehran, Iran‎.

**Keywords:** *Gorgan*, *Health Centers*, *Marital Satisfactions*, *Women*

## Abstract

**Objective: **One of the most important goals of marriage is the will to attain marital satisfaction. Marital ‎satisfaction is of prime importance and has the highest effect on the stability and failure of the ‎marriage. This study aimed to investigate marital satisfaction in the women who referred to ‎Gorgan Health centers.‎

**Method: **This was a descriptive-analytical study conducted in Gorgan. Two hundred seventy married ‎women who referred to health centers were selected using multi stage sampling method. ‎Data were collected using a reliable and valid questionnaire and analyzed using SPSS 18.‎

**Results: **The mean age of the participants was 33.39 ± 9.25 years. Marital satisfaction was in semi-desirable and desirable level in 66.3% and 33.7% of the participants, respectively. A significant ‎relationship was found between marital satisfaction and educational level of the participants (P<0.0001 χ^2^ = 32.09), educational level of the husband (P = 0.002 χ^2^ = 19.44), and occupation ‎of the husband (P<0.0001 χ^2^ = 19.17.‎

**Conclusion: **Marital satisfaction of the participants was in desirable level. We recommend offering an ‎appropriate intervention to promote marital satisfaction particularly based on the mentioned ‎variables. ‎

One of the most important goals of marriage is the will to attain marital satisfaction. Marital ‎satisfaction is the most important concern in marital life and has the highest effect on the ‎stability and failure of the marriage (1). On the other hand, some studies found an association ‎between marital dissatisfaction and different disorders such as depression (2) and health ‎problems during pregnancy (3). In addition, a study revealed that marital satisfaction in couples ‎with more traditional attitudes toward gender roles were significantly less than others (4). ‎Another finding indicated the significance of family processes (family coherence, problem-‎solving skills, communication skills and religious beliefs), social support and husband's ‎psychiatric disorders in marital satisfaction of women with major depressive disorder (5). In ‎addition, a study showed that marital satisfaction in parents of children with attention deficit ‎hyper activity disorder is lower than parents with normal children (6). Thus, to promote marital ‎satisfaction, we should evaluate marital satisfaction status and design interventional programs ‎based on the its associating factors. Considering the importance of marital satisfaction and its ‎associating factors and the unique socioeconomic and cultural condition in different ‎populations, this study aimed to investigate marital satisfaction in refereed women to Gorgan ‎Health centers.‎

## Materials and Method

This was a descriptive-analytical study conducted in 2013, in which 270 married women who ‎referred to Gorgan health centers were selected using multi stage sampling method. Data were ‎collected using a valid and reliable questionnaire and ENRIXH 47 questions marital satisfaction ‎scale (6 - 8). Scores were classified as follows: Scores between 0-24% as low (undesirable), 25-‎‎74% as moderate (semi desirable) and 75-100% as high (desirable) marital satisfaction. This ‎questionnaire obtained the demographic information of the participants including age, length of ‎marriage, level of education, occupation and marital satisfaction. Data were analyzed using ‎SPSS 18 software and chi-squared test.‎‎

## Results

The mean age of the participants was 33.39±9.25. The mean number of years married was ‎‎12.66±9.34 in women. Most participants were homemakers (85.2%). The educational level of ‎most participants was diploma (43%); most of their husbands were self-employed (48.9%) and ‎most husbands held a high school diploma (35.6%). Marital satisfaction was in semi- desirable ‎and desirable level in 66.3% and 33.7% of the participants, respectively. A significant ‎relationship was found between marital satisfaction and educational level of the participants (P<0.0001 χ^2^ = 32.09), educational level of the husband (P = 0.002 χ^2^ = 19.44), and occupation ‎of the husband (P<0.0001 χ^2^ = 19.17). Therefore, marital satisfaction was higher in people with ‎higher education and among the employed. However, no significant relationship was found ‎between marital satisfaction and participants’ occupation (P = 0.202 χ^2^ = 1.62) and length of ‎marriage (P = 0.260 χ^2^ = 4.1).‎ 

## Discussion

Marital satisfaction of the participants in this study was consistent with that of studies conducted ‎in Isfahan (9), Borojen (10), and Shahroud (11). However, marital satisfaction in Shiraz study ‎was higher than that of ours (12). Therefore, it seems status of marital satisfaction in different ‎parts of Iran is different, so more studies need to be conducted to determine factors contributing ‎to marital satisfaction, and appropriate interventions should be designed and offered based ‎on the socio-economic and cultural status of different parts of the country. ‎

Our findings revealed a relationship between marital satisfaction and educational level of the ‎participants and their husbands, which was consistent with Borojen study (10). Therefore, ‎marital satisfaction is higher among those with higher education. Thus, we should evaluate ‎marital satisfaction particularly in people with lower education level as a high-risk group and ‎provide them with appropriate interventions. ‎

Considering the association between marital satisfaction and educational level of the ‎participants, and educational level and occupation of their husbands, we recommend providing ‎appropriate interventions to promote marital satisfaction particularly based on the mentioned ‎variables.‎

## Limitations

Marital satisfaction was evaluated through a self- report questionnaire, so its precision may be in ‎question. Therefore, another study should be designed to evaluate marital satisfaction using ‎different methods such as private interviews.‎

## Conclusion

According to the findings, marital satisfaction of the under studied women was in desirable ‎level. Considering the association between marital satisfaction with the mentioned variables, we ‎recommend appropriate interventions to promote marital satisfaction, particularly based on the ‎mentioned variables.‎
